# Rising antifungal resistance in *Trichophyton* species—the bleak future for treatment of dermatomycosis?

**DOI:** 10.3389/fmicb.2026.1724650

**Published:** 2026-02-25

**Authors:** Anke Burmester, Jörg Tittelbach, Silke Uhrlass, Pietro Nenoff, Mario Fabri, Cornelia Wiegand

**Affiliations:** 1 Department of Dermatology, University Hospital Jena, Jena, Germany; 2 Labopart-Medizinische Laboratorien, Rötha OT Mölbis, Germany

**Keywords:** antifungal resistance, antimycotic therapy, dermatomycoses, multidrug-resistant fungi, *Trichophyton*

## Abstract

Dermatophyte infections, particularly those caused by *Trichophyton* species, represent a significant global health concern due to their high prevalence and increasing resistance to commonly used antifungal agents. While traditionally regarded as treatable with topical or systemic antifungals such as terbinafine and azoles, recent epidemiological shifts and misuse of antifungal medications have led to the emergence of multidrug-resistant strains, most notably *Trichophyton indotineae* (*T. mentagrophytes* subtype VIII). Resistance is often associated with genetic mutations in target enzymes and overexpression of efflux pumps. Inadequate treatment regimens, prolonged monotherapies, and combination with corticosteroids further exacerbate the selection of resistant isolates. Antifungal stewardship (AFS) is essential to combat resistance development. This includes targeted therapy based on mycological diagnostics, identification of the causative species, and appropriate patient education. Current antifungal therapies are limited to a few drug classes, and their efficacy is challenged by poor tissue penetration and subtherapeutic drug levels at infection sites. Innovative formulations and delivery systems may improve bioavailability and therapeutic outcomes. Combination therapies and the use of efflux pump inhibitors may offer additional options for recalcitrant infections. Ultimately, the growing resistance among *Trichophyton* species highlights an urgent need for novel antifungal agents, advanced diagnostics, and globally coordinated stewardship programs to safeguard the future of dermatomycosis treatment.

## 1 Introduction

The World Health Organization (WHO) has introduced the Global Antimicrobial Resistance and Use Surveillance System (GLASS), which also includes the GLASS-FUNGI module (Gupta et al., 2023a; World Health Organization, 2019). To date, the GLASS-Fungi module has focused exclusively on invasive fungal diseases as well as yeasts and non-dermatophytes, which are most frequently associated with these infections (Gupta et al., 2023b). Cutaneous fungal infections caused by dermatophytes have not yet been considered, although there is a clear need for action.

**Figure 1:**
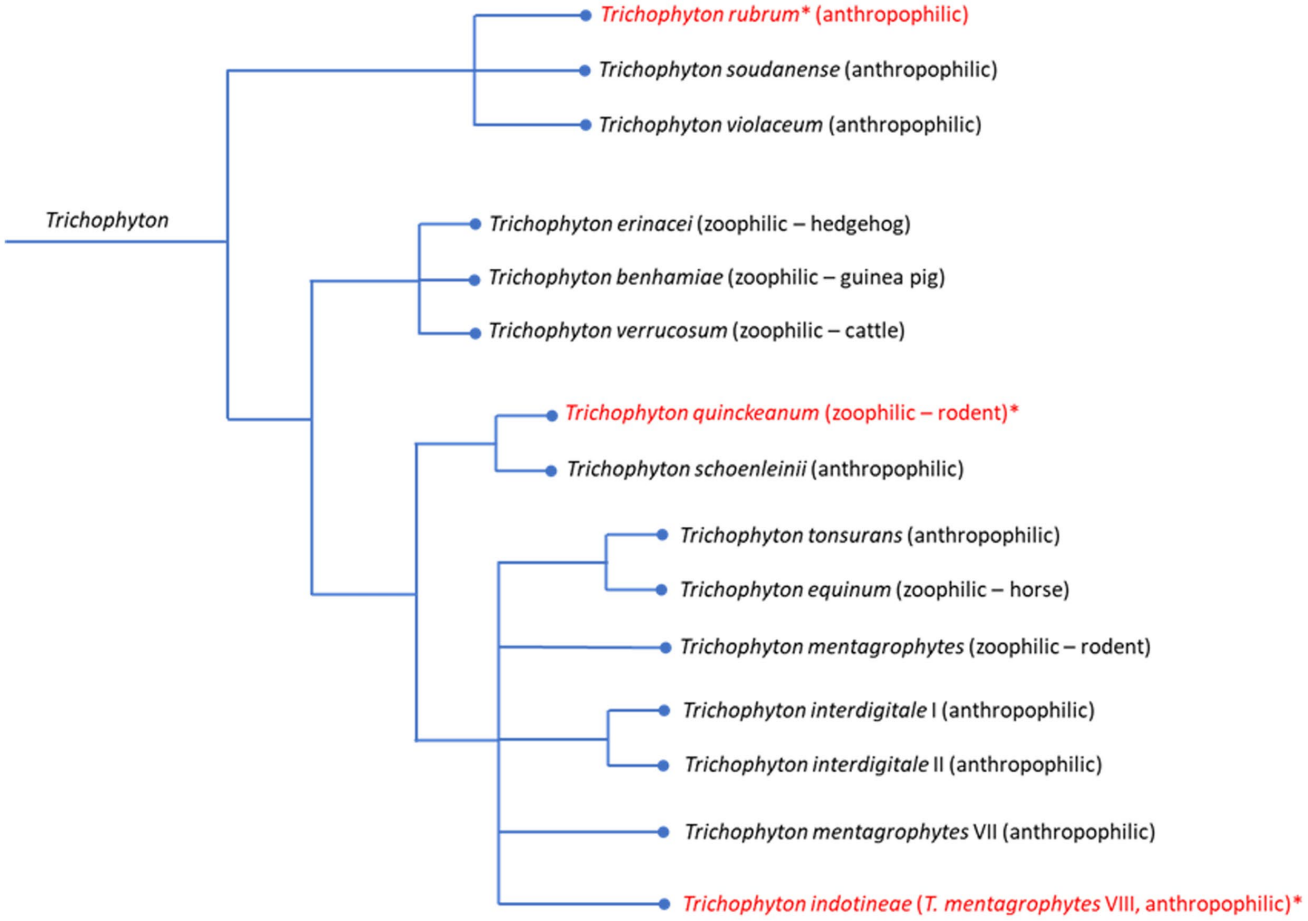
Schematic overview of phylogenetic relationships among *Trichophyton* species, including their epidemiological classification (anthropophilic, zoophilic, geophilic). Common animal reservoirs are indicated for zoophilic species. Species frequently associated with resistant isolates are marked in red with an asterisk (*).

In the past ten years, there has been a dramatically increased development of resistance in dermatophytes, especially in *Trichophyton* species (Figure 1). While older studies found only a small proportion of terbinafine-resistant isolates of the genus *Trichophyton* (Yamada et al., 2017), an outbreak in South Asia fundamentally changed this situation. In India, the previously most common dermatophyte pathogen *Trichophyton rubrum* was displaced by a novel pathogen from the *Trichophyton mentagrophytes/interdigitale* complex (Singh et al., 2018; Rudramurthy et al., 2018; Khurana et al., 2018; Nenoff et al., 2019). This pathogen is highly resistant to terbinafine and in some cases also shows reduced sensitivity to azoles. Typical infection patterns are tinea corporis and tinea cruris, often with a recurrent or therapy-resistant course (Nenoff et al., 2019). The molecular characterization was initially controversial. Genomic analyses showed that the pathogen with the D15P135 genome represents an independent lineage within the *T. mentagrophytes/interdigitale* complex (Pchelin et al., 2019). Due to the limited possibility of crossing with other strains, the new species name *Trichophyton indotineae* was introduced (Kano et al., 2020; Tang et al., 2021). This species is now distributed worldwide (reviewed in Jabet et al., 2023), including regular detections in Germany (Nenoff et al., 2020) and reported cases across Europe (Ferreira and Lisboa, 2025) and North America (Posso-De Los Rios et al., 2022; Caplan et al., 2023).

Exact species differentiation is essential for targeted therapy, as the *T. mentagrophytes/interdigitale* complex comprises several clinically relevant pathogens, including *T. interdigitale, T. mentagrophytes* (subtype VII) and *T. indotineae* (*T. mentagrophytes* subtype VIII), each of which can cause different infections. In addition, there are numerous other genetic variants within the complex illustrating the high diversity of this group of pathogens (Uhrlaß et al., 2022).

The development of resistance is presumably associated with the inappropriate use of antifungals and fixed combinations with glucocorticoids. In India in particular, creams containing potent glucocorticoids in combination with antifungal and antimicrobial agents are available over the counter (Bishnoi et al., 2018). These preparations are often cheaper than pure antifungals and are therefore widely available (Verma, 2018). Although corticosteroids relieve symptoms in the short term, they suppress the immune response and have hardly any antifungal effect. This leads to patients using these drugs for months or years without eliminating the infection. The result can be persistent, atypical skin mycoses with increased development of resistance (Verma and Madhu, 2017). A study from India with 402 patients showed that 78% of infections were due to *T. indotineae* (*T. mentagrophytes* subtype VIII), with 71% of isolates showing terbinafine resistance (Ebert et al., 2020). Furthermore, a study in the USA on 5,432 toenail samples revealed a mutation rate of 3.7% in *T. rubrum* and *T. mentagrophytes* associated with terbinafine resistance (Gupta et al., 2023a).

In view of these developments, it is urgently necessary to expand the WHO’s measures for cutaneous dermatophytoses. These must particularly include the systematic monitoring of resistant species, the expansion of laboratory capacities for resistance determination, the routine use of antimycograms and modern molecular diagnostics, the development of methods to identify resistance-causing mutations, and the promotion of rational antimycotic use at a global level (Gupta et al., 2023a).

## 2 Current trends in resistance development

### 2.1 Molecular mechanisms of terbinafine resistance

**Figure 2:**
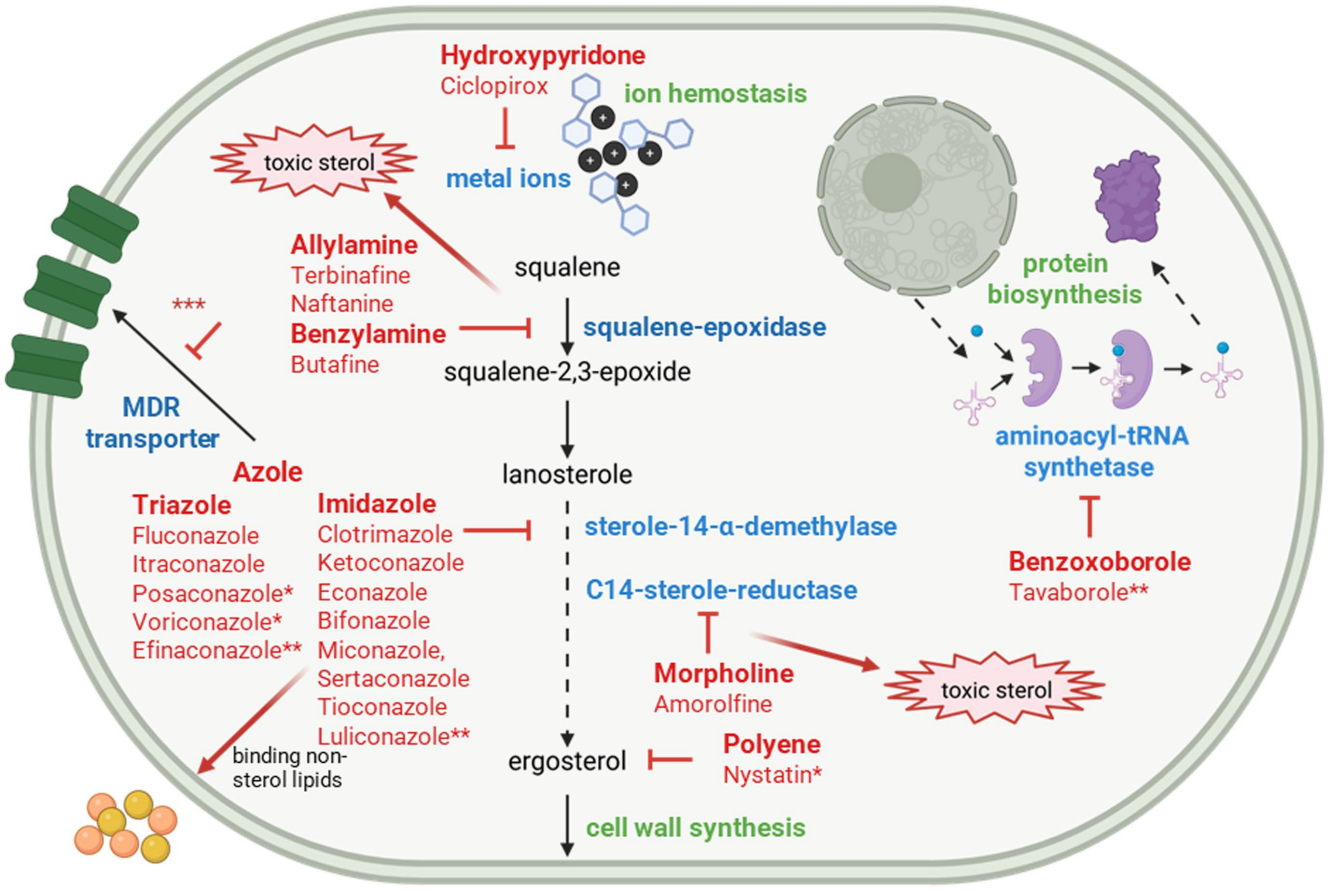
Schematic representation of key cellular processes in the fungal cell targeted by antifungal agents. Sites of action of established antifungal drugs are indicated; established drugs not approved for dermatomycosis are marked with an asterisk (*), novel compounds are denoted with a double asterisk (**) and new potential target sites are demonstrated with three asterisks (***) (Created with Biorender.com).

Most drugs used to combat fungal infections target the ergosterol biosynthesis pathway. Ergosterol is a vital component of fungal cell membranes. The inhibition of various steps in the biosynthetic pathway can massively reduce the growth of fungi and lead to cell death with the formation of toxic intermediates. Terbinafine binds to the enzyme squalene epoxidase and in this way interrupts the ergosterol metabolic pathway (Figure 2). The first terbinafine-resistant dermatophytes showed point mutations in Erg1, the gene for squalene epoxidase (Table 1). The amino acid changes in position Leu393Phe and Phe397Leu led to terbinafine-resistant *T. rubrum* isolates (Osborne et al., 2005; Osborne et al., 2006). Following these individual cases, over 2000 clinical isolates from Switzerland were tested in a large study and terbinafine-resistant isolates were found in less than 1% of cases (Yamada et al., 2017). Most of the resistant isolates from this study were *T. rubrum* strains. In addition to the described changes in *Erg1*, other exchanges were identified (Leu393Ser/Phe, Phe397/Ile/Val, Phe 415Ile/Ser/Val and His440Tyr).

**Table 1:** Overview of key mutations and regulation mechanisms of antifungal resistance and frequency of variants.

Gene/target	Key mutation(s) or regulation mechanism(s)	*Trichophyton* strain(s)	Frequency of genetic variants	Notes	References
Terbinafine					
*Erg1* (squalene epoxidase)	Phe397Leu, Leu393Phe	*T. rubrum*	<1% of 2000 clinical isolates from Switzerland with mutations 8 of 18 clinical isolates from India belong to Phe397Leu	Strong resistance, first reported clinical cases; multiple other mutations sites identified. Clinical isolates from India showed increased mutant frequency	Osborne et al. (2005), Osborne et al. (2006), Yamada et al. (2017), and Ebert et al. (2020)
	Leu393Phe, Phe397Leu	*T. indotineae*	12/20 strains Phe397Leu and 8/20 Leu393Phe	First reported cases. High MIC values; Phe397Leu more prevalent and successful	Singh et al. (2018), Rudramurthy et al. (2018), and Khurana et al. (2018)
	Ala448Thr (alone)	*T. indotineae*	~ 21% of around 280 clinical isolates	MIC values ≤ 0.1 μg/ mL; often co-occurs with type II genomic amplification of *Erg11B*	Ebert et al. (2020), Yamada et al. (2023)
	Phe397Leu Phe397Leu + Ala448Thr (double mutation) Leu393Phe/Ser	*T. indotineae*	~ 54% clinical isolates ~ 10% clinical isolates ~ 5% clinical isolates	MIC values ~ 8 μg/mL for Phe 397Leu MIC value of 16 μg/ mL for Leu393Phe, 0.5–1 μg/mL for Leu393Ser. double mutants associated with azole resistance	Ebert et al. (2020) and Burmester et al. (2022)
Azoles (itraconazole, voriconazole, fluconazole)					
*Erg11A/Erg11B* (sterol 14-α demethylase)	Ala230Thr, Asp441Gly, Gly443Glu, Tyr444His/Cys, Asp441Tyr, Gly443Arg, Tyr444Ser, Gly445Ser/Asp	*T. indotineae*	Tyr444His most often found	Mutations mostly in conserved motif DYGYG (positions 441–445 in Erg11B; difficult phenotype–genotype correlation)	Bhuiyan et al. (2024) and Burmester et al. (2022)
	Type I amplification (5–7 copies, all Gly443Glu)	*T. indotineae*	3 isolates	MIC values of 0.5–1 μg/ mL. Amplification occurred after point mutation; combination of overexpression + altered protein	Yamada et al. (2022) and Krauße et al. (2025)
	Type II amplification (7.4 kb fragment including Erg11B + neighbors)	*T. indotineae*	31/33 *Erg11B* type II isolates carry the *Erg1* Ala448Thr exchange	MIC values of 0.1–1 μg/mL. Overexpression is the main resistance mechanism	Yamada et al. (2023), Berstecher et al. (2024), and Krauße et al. (2025)
	Tyr136His Gly443Cys	*T. rubrum*	Single cases	Genetic variants isolated from each patient; difficult phenotype–genotype correlation	Kano et al. (2022) and Krauße et al. (2025)
Drug efflux/transporters					
*MDR/MFS* transporters	*MDR3, MDR2* overexpression in resistant isolate	*T. rubrum*		*MDR3* knockout reduces resistance	Monod et al. (2019)
	*MDR3* overexpression	*T. indotineae*		Strong *Erg11B* overexpression often sufficient; *MDR3* may act independently	Yamada et al. (2022) and Berstecher et al. (2024)
	*MFS1, MDR2, MDR3* overexpression	*T. mentagrophytes* (zoophilic)		Transporter overexpressionn upon drug exposure; transporter-mediated resistance	Gnat et al. (2021)
	*MDR*-like genes upregulation	*T. tonsurans*		Revealed by RNA-seq; efflux-based mechanism	Sammadar et al. (2025)

The first studies on *T. indotineae* showed comparable mutations in the *Erg1* gene (Rudramurthy et al., 2018; Singh et al., 2018; Khurana et al., 2018). Phe397Leu mutations were found in the *Erg1* gene of *T. indotineae* as well as Indian *T. rubrum* strains (Rudramurthy et al., 2018). In another study, 12 strains with Phe397Leu mutations and 8 strains with Leu393Phe mutations of *Erg1* were found among 20 resistant *T. indotineae* isolates (Singh et al., 2018). Both mutations lead to strong resistance to terbinafine, with high minimum inhibitory concentration (MIC) values (Singh et al., 2018; Rudramurthy et al., 2018; Khurana et al., 2018). However, strains with mutations in position 397 are more successful and in other studies, only a significantly low proportion of 3% each of resistant strains carrying Leu393Phe or Leu393Ser mutations was found (Ebert et al., 2020). In three major regions of India, over 70% of *T. indotineae* strains were resistant to terbinafine and only in the south of India was the proportion of sensitive strains higher than that of resistant strains (Ebert et al., 2020). Among the resistant strains, about 90% carried Phe397Leu mutations and about 14% carried another Ala448Thr mutation, which occurs as a double mutation in a certain proportion of strains (Ebert et al., 2020).

The resistance mechanism of terbinafine has been investigated for *Saccharomyces cerevisiae* using a three-dimensional squalene epoxidase protein model with terbinafine bound to it, which shows the close contact of the amino acid in position Phe402 with terbinafine (Nowosielski et al., 2011). *S. cerevisiae* strains with Phe402Leu mutations are also terbinafine-resistant (Leber et al., 2003) and this position corresponds to Phe397 in *Trichophyton* isolates. Comparison of the squalene epoxidase of *Trichophyton* isolates with those of other fungi shows the similarity of the comparable structural elements of *α*-helices and *β*-sheet structures at the structural level of the proteins (Sagatova, 2021).

In the meantime, screens for molecular alteration of the *Erg1* gene have identified further positions in *Erg1* that influence terbinafine resistance (Saunte et al., 2019; Astvad et al., 2022). Altered *Erg1* genes were found in *T. interdigitale* (Saunte et al., 2019; Moreno-Sabater et al., 2022) and in *T. mentagrophytes* isolates, the latter originating from human infections that were infected by different animals as sources (fox, guinea pig, dog, cat) (Gnat et al., 2021).

Interestingly, the second most common mutation in *Erg1* Ala448Thr of *T. indotineae* isolates does not lead to any significant increase in terbinafine resistance (Ebert et al., 2020; Burmester et al., 2020). However, a significant proportion of these strains show high tolerance to azoles (Ebert et al., 2020; Burmester et al., 2020). In strains carrying a double mutation (Phe397Leu, Ala448Thr), the proportion of azole-resistant isolates is even higher (Burmester et al., 2020). This indicates that the combination of high terbinafine resistance with azole resistance favors the selection of these double mutants and explains the difficulty of treatment up to treatment failure. In *T. indotineae*, the presence of *Erg1* Ala448Thr mutations can be used as a diagnostic marker to test for azole resistance in such isolates.

The commercially available test DermaGenius Resistance multiplex RT-PCR (TECOmedical Group, Rheinbach, Germany) is now available for the identification of the most important *Erg1* mutations (Leu393 and Leu397), which can identify mutated isolates from the T*. mentagrophytes/interdigitale* complex and *T. rubrum* using quantitative multiplex PCR (qPCR) (Sacheli et al., 2024; Duus et al., 2025). Diagnostic tools on a molecular basis have the advantage of being able to be used without culture, with DNA from scales (Burmester et al., 2019). They are able to provide a result at a very early stage so that the treatment regimen can be adapted accordingly. However, a low DNA concentration directly from patient material is limiting, as it can lead to an incorrect mutation analysis in the RT-PCR (Bhuiyan et al., 2024). Moreover, a negative molecular result does not rule out resistance based on other mechanisms, e.g., via activated drug efflux.

### 2.2 Molecular mechanisms of azole resistance

Azoles bind to sterol 14-*α* demethylases and suppress the enzymatic activity of these proteins so that ergosterol biosynthesis is interrupted at a later step (Figure 2). The genes are designated either according to the *Candida* nomenclature *Erg11* or according to their affiliation to the cytochrome P450 proteins *CYP51* (Song et al., 2018). Like *Aspergillus fumigatus*, *Trichophyton* species have two paralogous genes A and B (Mellado et al., 2001), while *Saccharomyces cerevisiae* has only one *Erg11* copy. *Trichophyton Erg11A* and *B* have been named after their similarity to the corresponding *Aspergillus* genes. In *Aspergillus*, the changes in the sterol 14-α demethylase genes are based on two main mechanisms (Dudekova et al., 2017). The first possibility consists of point mutations of the gene that lead to altered amino acids. The binding of azoles is impaired in these altered proteins. Depending on the chemical structure of the azoles and their interaction with the sterol 14-α demethylases, different amino acid changes prevent the binding of different azole classes (Rosam et al., 2021). The second pathway involves the overexpression of genes, which lead to increased protein levels that can bind the administered azoles while still maintaining sufficient enzymatic activity in ergosterol metabolism (Dudekova et al., 2017). Both pathways are also present in *Trichophyton indotineae* and even overlap, so that an estimation of the resistance potential of the strains can be difficult.

Sequencing of the two *Erg11* paralogous genes in *T. indotineae* revealed that amino acid exchanges are concentrated on *Erg11B* (Burmester et al., 2022). In the first strains, exchanges were found in positions Ala230Thr, Asp441Gly, Gly443Glu, Tyr444His/Cys partly as double mutations (Burmester et al., 2022). An overview is given in Table 1. Further *Erg11B* amino acid substitutions were identified in *Erg11B* genes of *T. indotineae* isolates from Bangladesh, with the following additional variants Asp441Tyr, Gly443Arg, Tyr444Ser, Gly445Ser/Asp (Bhuiyan et al., 2024). Most mutations are concentrated in a protein motif conserved in both paralogous *Erg11* genes in position 441–445 in Erg11B and in 431–435 in Erg11A with the sequence Asp-Tyr-Gly-Tyr-Gly (in single-letter code DYGYG). This motif is also conserved in other fungi and in both paralogous *Erg11* genes of *A. fumigatus* (Burmester et al., 2022). In the yeasts *Candida* and *Saccharomyces* there is a variation in this motif in the second tyrosine to a phenylalanine (DYGFG). Interestingly, resistant *Candida* strains show *Erg11* mutations with comparable changes in this motif (Asp446Asn, Tyr447Gly/His, Gly448Glu/Arg/Val, Phe449Leu/Ser/Val/Ile, Gly450Glu/Val) (Becher and Wirsel, 2012). Resistant *A. fumigatus* strains also show exchanges in this motif of Erg11A (Tyr431Cys, Gly434Cys) (Becher and Wirsel, 2012). Phytopathogenic fungi that have adapted to azoles used in agriculture also show changes in this motif (Becher and Wirsel, 2012; Brunner et al., 2008). Azoles in agriculture have an impact on increasing rates of resistant *A. fumigatus* strains and the control of invasive mycoses (Meis et al., 2016). The zoophilic skin fungus *Trichophyton quinckeanum* showed a new genotype with an altered *Erg11A* gene and increased itraconazole resistance compared to earlier variants (Winter et al., 2023). The number of infections in humans correlates with the population of field mice (Winter et al., 2023) and is transmitted via hunting cats (Gregersen et al., 2021). Azoles used in agriculture may also play a role in the displacement of sensitive genotypes in the microbiome of wild animals. The phylogenetic analysis of *T. indotineae* (Pchelin et al., 2019) shows the similarity to zoophilic *T. mentagrophytes* subtypes. It should therefore be considered that resistance to agriculturally used azoles has developed in a previously unknown animal reservoir of *T. indotineae*. This would explain why some of these mutated *Erg11B* variants are not resistant to azoles used for medicinal purposes (Bhuiyan et al., 2024).

It is also difficult to correlate the phenotype and genotype in *T. indotineae* because overexpression of *Erg11B* also leads to azole resistance (Yamada et al., 2022). The overexpression of *Erg11B* is caused by the amplification of genomic *Erg11B* copies (Yamada et al., 2022). In type I amplification (Table 1), five to seven copies of *Erg11B*, as repetitive elements of a 2.4-kb DNA fragment, were arranged in sequence in the same orientation (Yamada et al., 2022). Interestingly, all these copies carry a point mutation that leads to Gly443Glu exchange. Similarly, all genomes of type I show the same point mutation in *Erg11B*. This is a combination of overexpression of *Erg11B* producing altered *Erg11B* proteins. It can be concluded that the amplification occurred after the point mutation occurred and developed from a mutant isolate with one copy. A *T. indotineae* Gly443Glu isolate with only one copy already showed an approximately fourfold increase in IC values against itraconazole (Burmester et al., 2022). Interestingly, a three-dimensional model of *A. fumigatus* Erg11A protein by artificially introducing mutations identified positions that may be important for itraconazole binding (Liu et al., 2016). According to this model, the *A. fumigatus Erg11A* position Gly432, which corresponds to *T. indotineae Erg11B* Gly443, is involved in the binding of itraconazole (Liu et al., 2016). The itraconazole resistance of strains with type I amplification is even higher as it is based on two mechanisms. Moreover, these strains show increased resistance to voriconazole (Yamada et al., 2022).

A second type of amplification of *Erg11B* was discovered (Table 1), which was initially overlooked even in genomes decoded at the highest level (Yamada et al., 2023). In this case, a 7.4 kb DNA fragment is amplified, which, in addition to *Erg11B*, also contains the right and left neighboring genes (Yamada et al., 2023). This type is more common and correlates with the *Erg1* Ala448Thr mutation. *Erg11B* indicates wild-type sequences and the resistance effect is mainly due to the overexpression of *Erg11B*. A multidrug-resistant strain UKJ 476/21 from the University Hospital Jena also showed high IC values against fluconazole, voriconazole and itraconazole (Burmester et al., 2022). Here, overexpression of *Erg11B* was the underlying mechanism of resistance (Berstecher et al., 2024). After further analysis, this strain belonged to the group of type II genomic amplification of Erg11B (Krauße et al., 2025).

Under heat-stress-inducing conditions, sensitive *T. indotineae* isolates also showed increased expression of *Erg11B*, even when *Erg1* and *Erg11A* expression fell below the detection limit so that new synthesis of ergosterol is unlikely (Berstecher et al., 2024). This suggests a stress-induced expression of *Erg11B* associated with a different function independent of ergosterol biosynthesis. In *A. fumigatus*, the two sterol 14-*α* demethylases differ in their substrate specificity (Hargrove et al., 2015). *A. fumigatus* Erg11A can convert both lanosterol and eburicol, whereas *A. fumigatus* Erg11B can only use eburicol as a substrate (Hargrove et al., 2015). These differences can be seen from the three-dimensional model and the identified binding pocket (Hargrove et al., 2015). The differences found in this domain are also evident in both T*. indotineae* paralogous *Erg11* genes, such that *T. indotineae Erg11B* resembles an Eburicol 14-α demethylase. In *A. fumigatus*, the eburicol level was identified as an important factor causing toxic effects in *A. fumigatus* upon accumulation of the same (Elsaman et al., 2024). The function of *T. indotineae Erg11B* may lie in the regulation of eburicol in order to minimize toxic intermediates of the ergosterol metabolism. Interestingly, the two co-amplified genes of *Erg11B* have functions in UV damage repair and in the regulation of telomere length (Yamada et al., 2023).

After identifying both amplification types, they can be analyzed in *T. indotineae* isolates using simple PCR and agarose gel electrophoresis, serving as a diagnostic tool (Yamada et al., 2023). For adapting the method to other *Trichophyton* species, qPCR with genomic DNA instead of cDNA proves more effective, as the recombination sites potentially involved in *Erg11B* amplification cannot be predicted. This method was also used to determine the copy number of both *T. indotineae* amplification types (Yamada et al., 2023). For type I, the copy numbers in the genomes were nearly identical to the qPCR data (Yamada et al., 2023). In type II genomes, however, the copy number of *Erg11B* was significantly underrepresented and differed greatly from the qPCR data, which indicated much higher copy numbers (Yamada et al., 2023).

Multidrug resistance, in the form of combined terbinafine and azole resistance, has so far only been found in isolated cases caused by *T. rubrum* (Kano et al., 2022; Nenoff et al., 2023). A common feature of these cases is a very long treatment period spanning years to decades in elderly individuals with chronic dermatomycosis. In both cases, genetically distinct *T. rubrum* isolates were obtained from a single patient. The *T. rubrum* isolate from Japan exhibited an *Erg1* Phe397Leu point mutation and an *Erg11B* Tyr136His mutation (Kano et al., 2022). This *Erg11* mutation is well-known in *A. fumigatus* and many other fungi, and its selection is associated with the use of the short-chain azole voriconazole (Rosam et al., 2021). In these fungi, the Tyr136His mutation leads to resistance to voriconazole, but not to itraconazole (Rosam et al., 2021). In the second case, an unusual *Erg1* Ile479Thr mutation was identified in *T. rubrum*, which results in increased terbinafine resistance (Nenoff et al., 2023). This *T. rubrum* strain also carries an *Erg11B* Gly443Cys mutation, which is associated with elevated IC values for itraconazole (Krauße et al., 2025).

### 2.3 Overexpression of transporters enables efflux of antifungal agents

An alternative mechanism that can reduce the effectiveness of antifungal drugs is the increased efflux of these compounds from the fungal cell (Figure 2). This mechanism, which also contributes significantly to drug resistance in humans, is mediated by transport proteins. Based on their structural similarity, these transporters are classified as either MDR (multidrug resistance) or MFS (major facilitator superfamily) transporters. Comparable transporters are found in many fungi (Table 1). For *Trichophyton*, they were first studied in detail in *Trichophyton rubrum* isolates. In *T. rubrum*, several genes encoding transporters involved in azole efflux have been identified, and some of these are overexpressed in azole-resistant strains (Monod et al., 2019). The *T. rubrum* MDR3 transporter can export both voriconazole and itraconazole, whereas the MDR2 transporter is specific for itraconazole (Monod et al., 2019). An *MDR3* knockout mutant in *T. rubrum* lost its resistance to voriconazole and showed reduced IC values for itraconazole (Monod et al., 2019). In resistant isolates, *MDR3* and *MDR2* were overexpressed compared to sensitive isolates (Monod et al., 2019). In *T. indotineae*, the situation is somewhat different. In some azole-resistant strains, *MDR3* was also overexpressed; however, other resistant strains expressed *MDR3* at similar levels to sensitive isolates (Yamada et al., 2022). Moreover, *MDR3* knockout mutants in *T. indotineae* did not show any reduction in resistance to either itraconazole or voriconazole (Yamada et al., 2022). The strong overexpression of *Erg11B* in *T. indotineae* alone is sufficient to confer resistance to both azoles. It remains possible that in strains lacking *Erg11B* overexpression, *MDR3* overexpression alone could mediate azole resistance, with both systems acting independently as “backup” mechanisms. Expression analyses of transporters in other *T. indotineae* strains show that basal levels of MFS1 transcripts are elevated in some isolates (Berstecher et al., 2024). In the azole-resistant *T. rubrum* isolate, the addition of itraconazole or voriconazole led to an increased expression of *MDR3* (Monod et al., 2019). In *T. indotineae*, *MDR3* expression could be induced in both sensitive and resistant isolates, primarily by fluconazole and, to a lesser extent, by voriconazole, while itraconazole and terbinafine had little effect (Berstecher et al., 2024). All *T. indotineae* isolates show high tolerance to fluconazole, regardless of their ability to overexpress *Erg11B*, and activation of *MDR3* may be a possible explanation for this observation.

Studies of *T. mentagrophytes* isolates transmitted from animals such as foxes or guinea pigs to humans revealed several terbinafine-resistant isolates that did not carry the corresponding *Erg1* mutation (Gnat et al., 2021). Often, isolates with or without the *Erg1* Phe397Leu mutation also exhibited elevated IC values against azoles (Gnat et al., 2021). In isolates lacking *Erg1* mutations, resistance must be mediated via an alternative mechanism. Expression analyses of the resistant *T. mentagrophytes* isolates showed increased *MFS1* expression following the addition of terbinafine or itraconazole, whereas *MDR3* and *MDR2* were only induced by itraconazole (Gnat et al., 2021). These findings more closely resemble the pattern observed in *T. rubrum* (Monod et al., 2019). Recently, *T. tonsurans* isolates exhibiting multidrug resistance to various azoles and terbinafine have been linked to the up-regulation of multiple *MDR*-like genes, as revealed by RNA-seq analysis (Sammadar et al., 2025). In *T. rubrum* and in *T. mentagrophytes*, transporter inhibitors were able to enhance sensitivity to itraconazole or voriconazole (Gnat et al., 2021; Monod et al., 2019). Such inhibitors could potentially be used in combination with azoles or terbinafine to improve future treatment strategies. However, further research is needed for diagnostics at the transporter level to better understand the highly specific responses across different *Trichophyton* species.

## 3 Dermatomycological diagnostics and resistance testing

At first glance, taxonomic classifications of fungi may seem distant from clinical practice; however, taxonomy plays a crucial role in several respects. Accurate diagnostics before the start of treatment represent the first and decisive step toward successful therapy. Different dermatophyte species exhibit significantly varied susceptibility profiles to antifungal agents (Gräser and Saunte, 2020; Begum et al., 2020). Therefore, species-specific identification is essential for selecting the appropriate antifungal therapy. It not only informs optimal treatment strategies but also provides insights into the source of infection, an important factor for outbreak management and prevention of reinfection (Gräser and Saunte, 2020).

**Figure 3:**
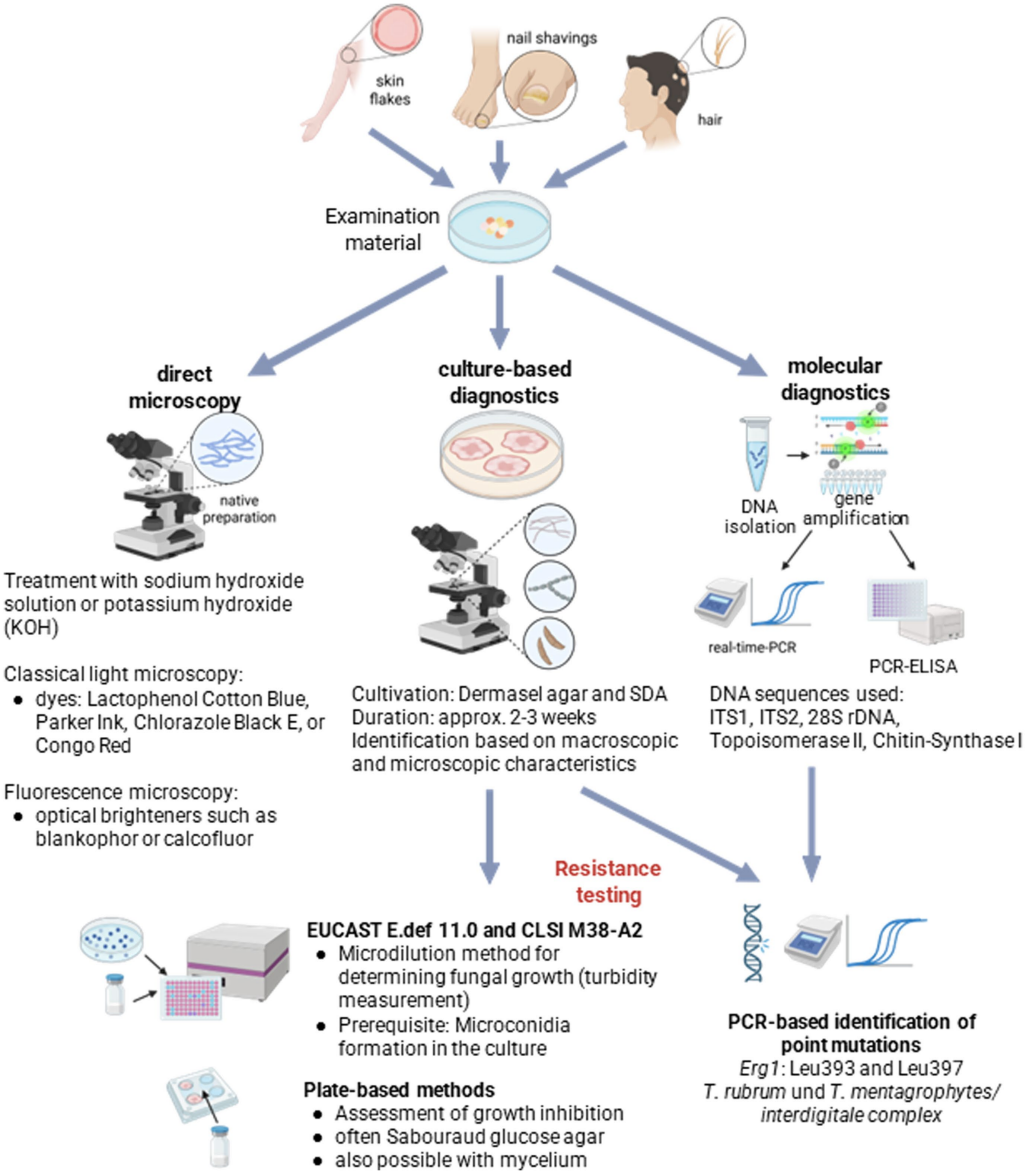
Overview of the diagnostic approach in fungal infections for confirmation of the suspected diagnosis and identification of the causative organism, including available methods for antifungal susceptibility testing (created with BioRender.com).

The species name can offer clinical clues regarding the infection source, whether it originated from a human, animal, or the environment. Identifying the source allows for targeted treatment of the index patient or affected animal, helping to prevent further spread of the infection (Gräser and Saunte, 2020). Typing at the subspecies level is also critical for epidemiological investigations. This was evident in India, where genotype VIII of *Trichophyton mentagrophytes* was identified as the causative agent of a nationwide epidemic of chronic, recurrent dermatophytoses. In-depth study of this genotype provided important insights into virulence factors and resistance mechanisms (Jabet et al., 2023). Furthermore, precise mycological diagnostics are necessary to distinguish dermatophytes from other pathogens. Molds such as *Fusarium* or *Aspergillus*, as well as yeasts like *Candida* and *Malassezia*, require entirely different therapeutic approaches than dermatophytes (Petrucelli et al., 2020). Even a negative fungal result has clinical significance, as it helps rule out fungal infections and prompts consideration of other dermatological diagnoses (Gräser and Saunte, 2020). The combination of classical and molecular diagnostic methods (Figure 3) significantly improves pathogen identification, enables targeted therapy, supports early detection of resistance, and ultimately contributes to optimizing treatment outcomes (Gupta et al., 2023b; Gräser and Saunte, 2020; Begum et al., 2020; Petrucelli et al., 2020).

For the initial identification of fungal elements in clinical samples, direct microscopy is commonly employed (Figure 3, upper left). In this method, specimens are treated with sodium hydroxide or potassium hydroxide (KOH) to prepare a wet mount. However, while this approach allows for the visualization of fungal structures, it does not permit further identification at the genus or species level. To enhance detection sensitivity, fluorescence microscopy can be used in addition. Here, samples are treated with optical brighteners such as Blankophor or Calcofluor, which bind to fungal cell walls and make fungal elements more visible under UV light.

Despite its limitations, fungal culture (Figure 3, middle) remains the gold standard in medical mycology and is the standard method for identifying dermatophytes (Kidd and Weldhagen, 2022). Culture confirms the viability of the pathogen (Gräser and Saunte, 2020; Gnat et al., 2020) and allows for the isolation of pure strains that can be used for downstream analyses such as resistance testing. However, culture is time-consuming, and its sensitivity varies widely, ranging from 23.8 to 79.3%, depending on various factors (Kidd and Weldhagen, 2022; Chanyachailert et al., 2023). False-negative results are common and often caused by external influences such as prior topical antifungal treatment or inadequate sample collection (Gräser and Saunte, 2020; Begum et al., 2020; Petrucelli et al., 2020). Moreover, mixed infections and morphologically similar species, particularly within the *T. mentagrophytes* complex, further complicate accurate diagnosis (Gupta et al., 2023b).

Molecular techniques (Figure 3, upper right) offer a valuable complement to classical diagnostic methods and significantly improve therapeutic planning in cases of dermatophytosis (Begum et al., 2020; Petrucelli et al., 2020; Kidd and Weldhagen, 2022; Chanyachailert et al., 2023). Particularly in light of the increasing spread of resistant dermatophytes, they also contribute substantially to epidemiological studies. Compared to culture-based methods, molecular diagnostics are characterized by significantly higher sensitivity and specificity, as well as much faster turnaround times. PCR-based methods and DNA sequencing, in particular, allow for rapid and reliable pathogen identification (Gupta et al., 2023a). Moreover, molecular biological techniques are essential for the precise differentiation of genetically closely related species, such as those within the *T. mentagrophytes* complex and *T. interdigitale* (Gupta et al., 2023b). Despite these advantages, molecular methods are not without limitations. Many PCR-based test systems are designed to detect only a limited number of species and may not distinguish between contaminating fungi and true pathogens (Gupta et al., 2023b; Gräser and Saunte, 2020; Kidd and Weldhagen, 2022). In addition, factors such as sample preparation, the presence of inhibitors in the specimen, selection of appropriate primers, and DNA quality can all significantly affect the accuracy of results (Begum et al., 2020). Nevertheless, numerous studies have shown that PCR-based techniques can increase the detection rate by up to 30% compared to culture-based methods (reviewed in Begum et al., 2020; Kidd and Weldhagen, 2022).

The increasing occurrence of resistance in dermatophytes, particularly to terbinafine, can necessitate targeted susceptibility testing. Clinical resistance is defined as the persistence of a fungal infection despite adequate therapy (Gupta et al., 2023b; Gnat et al., 2020). Both phenotypic and molecular genetic methods are employed for resistance testing.

Molecular techniques allow for the targeted detection of mutations in genes encoding the antifungal drug targets (Figure 3, lower right), such as *ERG1* (squalene epoxidase) in the case of terbinafine resistance, or *ERG11* (sterol 14-*α*-demethylase) for azole resistance. For rapid detection of specific *ERG1* mutations, real-time PCR-based assays are already available (Sacheli et al., 2024; Duus et al., 2025). However, phenotypic resistance testing remains indispensable, as molecular techniques cannot definitively rule out resistance in the absence of detected mutations. A critical prerequisite for phenotypic testing is the successful isolation of viable pathogens via culture, a process that may be compromised if samples are taken during ongoing antifungal treatment.

Two established methods exist for determining the minimum inhibitory concentration (MIC) in dermatophytes: the CLSI and the EUCAST methods (Figure 3, lower left). Both are based on measuring turbidity as an indicator of fungal growth compared to a reference standard (Gupta et al., 2023a). The microdilution method is considered the gold standard, provided the isolate has produced a sufficient number of microconidia. For this purpose, the general EUCAST protocol E.def 9.4 for pathogenic fungi as well as the specifically adapted protocol E.def 11.0 for *Trichophyton* isolates are available (Arendrup et al., 2024; Arendrup et al., 2021; CLSI, 2008). The main differences between the EUCAST method (E.def 11.0) and the CLSI method (M38-A2) concern mainly the incubation temperature and the spore concentration used, which is significantly lower in the CLSI method. Therefore, the CLSI method can offer advantages particularly with isolates exhibiting low sporulation. Evaluation is carried out either visually or photometrically. A more precise and automated measurement is enabled by microplate laser nephelometry, which performs continuous measurements and generates complete growth curves (Burmester et al., 2022; Winter et al., 2023; Fink et al., 2022). However, standardized clinical MIC breakpoints that clearly differentiate wild-type strains from resistant isolates currently do not exist (Gupta et al., 2023b; Sacheli and Hayette, 2021). Within the EUCAST method, at least preliminary epidemiological cut-off values (ECOFFs) have been defined. For example, an MIC of ≥ 0.06 mg/L for *T. rubrum* is considered indicative of terbinafine resistance associated with mutations in the *ERG1* gene (Gupta et al., 2023b). Another option for MIC determination are strip-based tests with concentration gradients of the active substances (Sacheli et al., 2024). However, this method also requires a standardized microconidia suspension and is thus dependent on the sporulation ability of the isolate, similar to the microdilution method. Since these procedures are complex, costly, and dependent on sufficient spore formation, they are only partially suitable for routine diagnostics. Therefore, plate-based methods have been developed for a more pragmatic estimation, where growth inhibition is assessed depending on the applied drug concentration (Bhuiyan et al., 2024; Winter et al., 2023; Blanchard et al., 2023). These often use Sabouraud glucose agar, which, unlike RPMI1640-based media, is commercially available or easy to prepare in-house.

## 4 Treatment of infections with resistant *Trichophyton* species

Antifungal therapy must be guided by a confirmed laboratory diagnosis. Notably, up to 40% of clinical presumptive diagnoses, especially in onychomycoses, are incorrect (Effendy et al., 2005). Therefore, initiating oral therapy without prior pathogen confirmation is strongly discouraged. Besides potential side effects and drug interactions, particularly in older patients with comorbidities and polypharmacy, there is also the risk of negative impacts on the microbiome and the promotion of drug resistance, as is known from antibiotic therapy but equally applies to antifungals (Gräser and Saunte, 2020).

Treatment depends on the extent, severity, site of infection, and pathogen involved and is carried out with topical and/or oral antifungals (overview Figure 2; Table 2). Topical antifungal therapy forms the backbone of treatment for localized infections and is indicated for skin infections with limited spread. Systemic (oral) medications are used in cases of extensive infections, chronic courses, or failure of topical therapy and are generally recommended for nail infections (onychomycoses) (Nenoff et al., 2015). Combination therapy is considered to be more effective mycologically and clinically than the use of either systemic or topical agents alone (Nenoff et al., 2015). It is recommended to combine agents from different classes to cover a broader spectrum of activity and reduce the risk of resistance development (Jartarkar et al., 2021). The main antifungal classes are polyenes, azoles, and allylamines/benzylamines (Figure 2; Table 2).

**Table 2:** Antifungal agents for dermatophytoses: overview on mechanisms of action, routes of administration, treatment regimens, and possible adverse effects.

Antifungal class*/*drug	Mechanism of action	Primary target	Route of administration	Indication	Dose and treatment duration		Common side effects
					Adults	Children	
Allylamines							
Terbinafine	Inhibits squalene epoxidase, blocking ergosterol synthesis and causing toxic squalene accumulation, leading to membrane dysfunction and fungal cell death	Squalene epoxidase	Topical and systemic (oral)	Tinea corporis	Topical once daily 2–4 weeks	Same as adults	Itching and local irritation possible Gastrointestinal disturbances, taste disturbances, headaches Hepatotoxicity (liver function tests recommended) Drug interactions possible
				Tinea capitis	Systemic 250 mg/day, 4–8 weeks	Weight-adjusted, 4–8 weeks (off-label in Germany)	
				Onychomycosis	250 mg/day; fingernails up to 6 weeks, toenails up to 12 weeks	Individual therapeutic trial only	
Naftifine	Inhibits squalene epoxidase with fungicidal and anti-inflammatory activity	Squalene epoxidase	Topical only	Tinea corporis	Applied twice daily for a period of 2–4 weeks	Same as adults	Local skin reactions (burning, erythema, pruritus)
				Onychomycosis	Treatment durations of up to 6 weeks	Same as adults	
Azoles – Imidazoles							
Clotrimazole, Ketoconazole, Econazole, Bifonazole, Miconazole, Tioconazole	Inhibit ergosterol biosynthesis, fungistatic to partially fungicidal	Lanosterol 14α-demethylase	Topical only	Tinea corporis	1–2 × daily for 2–4 weeks	Same as adults	Local burning, itching, dry skin, erythema, contact dermatitis
Sertaconazole	Inhibits ergosterol biosynthesis and binds to non-sterol membrane lipids, increasing permeability; anti-inflammatory	Ergosterol synthesis and fungal membrane	Topical only	Tinea corporis	Once daily for 4 weeks	Same as adults	Mild local irritation: burning, itching, dry skin, erythema
Azoles – Triazoles							
Fluconazole	Inhibits ergosterol biosynthesis	Lanosterol 14α-demethylase	Systemic (oral); topical formulations available	Tinea corporis	100–200 mg/day, 4–8 weeks	5–6 mg/kg/day 8 mg/kg/week for 3–6 weeks or up to 12 weeks use in children ≥1 year if no alternatives exist	Gastrointestinal symptoms, headache; hepatotoxicity possible Interaction with other drugs (potent inhibitor of CYP2C9 and CYP3A4; overdosing of drugs that are metabolized via these enzyme systems may occur)
				Tinea capitis	100–200 mg/day, 4–8 weeks	5–6 mg/kg/day 8 mg/kg/week for 3–6 weeks or up to 12 weeks Use in children ≥1 year if no alternatives exist	
				Onychomycosis	150–300 mg weekly for 3–6 months (fingernail) or up to 12 months (toenail)	3–5 mg/kg/day (max 50 mg), continuously or with a short loading phase followed by weekly doses, until healthy nails regrow	
Itraconazole	Inhibits ergosterol biosynthesis	Lanosterol 14α-demethylase	Systemic (oral)	Tinea corporis	100–200 mg/day, 2–4 weeks	Off-label	Gastrointestinal disturbances, hepatotoxicity, edema, drug–drug interactions
				Tinea capitis	100–200 mg/day, 4–8 weeks	Off-label	
				Onychomycosis	Continuous 200 mg/day for 4–12 weeks or pulse therapy Pulse therapy: 200 mg twice daily for one week, followed by an interval of 3 weeks, generally 3 cycles	5 mg/kg/day for 6–12 weeks (attention: not approved for therapy in children in Germany)	
Benzylamines							
Butenafine	Inhibits squalene epoxidase, causing fungicidal squalene accumulation; anti-inflammatory	Squalene epoxidase	Topical only	Tinea corporis	Once daily 2–4 weeks	Same as adults	Mild local irritation, burning or itching, skin dryness
Hydroxypyridones							
Ciclopirox	Chelates divalent metal ions, inactivating metal-dependent enzymes and disrupting fungal metabolism	Metal-dependent fungal enzymes	Topical only (nail lacquer, creams, ointments)	Tinea corporis	1–2 × daily 2–4 weeks	Same as adults	Burning, erythema
				Onychomycosis	Nail lacquer daily for several months	Same as adults	Nail discoloration
Morpholines							
Amorolfine	Inhibits Δ14-reductase and Δ7–Δ8-isomerase, leading to ergosterol depletion and toxic intermediate accumulation	Ergosterol biosynthesis enzymes	Topical only (mainly nail lacquer)	Onychomycosis	Nail lacquer 1–2 × weekly, 6–12 months	Same as adults	Local skin reactions: burning, erythema, itching, contact dermatitis Nail changes: nail discoloration, nail splitting (onychorrhexis)
Polyene							
Nystatin	Binds to ergosterol in the fungal cell membrane, forming pores and causing leakage of intracellular ions and molecules, ultimately leading to fungal cell death.	Ergosterol	Topical and systemic (oral)	Recommended for yeast and molds, *in vitro* efficacy against dermatophytes shown	-	-	Nausea, vomiting, diarrhea; topical/oral exposure can cause rash or hypersensitivity; high doses may be nephrotoxic.
Newer/alternative antifungals							
Posaconazole, Voriconazole (triazoles)	Inhibit ergosterol biosynthesis	Lanosterol 14α-demethylase	Systemic (oral*/*IV; off-label for dermatomycoses)	Strong *in vitro* activity against dermatophytes but only approved for invasive mycoses	Off-label, individualized	No recommendation	Hepatotoxicity, visual disturbances (voriconazole), gastrointestinal symptoms
Efinaconazole (triazole)	Inhibits ergosterol biosynthesis	Lanosterol 14α-demethylase	Topical only	Onychomycosis	Once daily up to 12 months	Same as adults; ≥ 6 years	Application-site dermatitis, erythema
Luliconazole (imidazole)	Inhibits ergosterol biosynthesis	Lanosterol 14α-demethylase	Topical only	Tinea corporis	Once daily 2–4 weeks	Same as adults; ≥ 12 years	Mild local irritation or pruritus
Tavaborole (benzoxaborole)	Inhibits aminoacyl-tRNA synthetase, blocking protein biosynthesis	Aminoacyl-tRNA synthetase	Topical only	Onychomycosis	Once daily up to 12 months	Same as adults; ≥ 6 years	Erythema, pruritus

The group of allylamines includes terbinafine and naftifine. Terbinafine inhibits squalene epoxidase, thereby interrupting ergosterol synthesis, which leads to membrane dysfunction and fungal cell death (Figure 2; Table 2). It is available both topically and orally and is used for superficial skin mycoses as well as nail mycoses (Gupta et al., 2023a; Nenoff et al., 2015; Sahni et al., 2018). Oral therapy duration depends on the species of the pathogen and the site of infection, typically ranging from 2 to 8 weeks, but can be longer in cases of onychomycoses (Nenoff et al., 2015). In cases of poor response, higher doses and/or extended treatment durations may be required (Gupta et al., 2023a; Sahni et al., 2018). Naftifine is also an allylamine with fungicidal activity (Figure 2; Table 2) against dermatophytes, *Candida*, and *Aspergillus*. It is used topically as a gel or cream for tinea pedis, cruris, and corporis, and additionally possesses anti-inflammatory properties that help alleviate symptoms (Sahni et al., 2018; Gupta et al., 2008; Stein Gold et al., 2015).

Azoles are divided into two main groups: imidazoles and triazoles. Imidazoles, such as clotrimazole, ketoconazole, econazole, bifonazole, miconazole, sertaconazole, and tioconazole (Nenoff et al., 2015), are applied topically in creams, ointments, and solutions and exert both fungistatic and partially fungicidal effects by inhibiting ergosterol biosynthesis (Figure 2; Table 2). Sertaconazole additionally binds to non-sterol lipids in the fungal membrane, increasing its permeability and acting against dermatophytes and *Candida*. Its anti-inflammatory properties improve patient compliance (Croxtall and Plosker, 2009; Gupta et al., 2017). Triazoles also inhibit ergosterol biosynthesis (Figure 2; Table 2). This group includes fluconazole and itraconazole, which can be used both topically and orally (Nenoff et al., 2015). Fluconazole shows lower efficacy against dermatophytes than itraconazole or terbinafine and is approved in Europe for dermatomycoses, but not in the USA (Gupta et al., 2023a). However, it should not be used in children under 1 year of age and only if no therapeutic alternative is available in children under 16 years (Nenoff et al., 2015). Nevertheless, itraconazole and terbinafine, for example, are not approved for therapy in children in Germany, so treatment is only possible as an individual therapeutic trial with parental consent according to the Medicines Act (Nenoff et al., 2015). Oral therapy with itraconazole can be administered continuously or as pulse therapy over several weeks to months, especially in onychomycoses (Gupta et al., 2023a; Nenoff et al., 2015).

In addition to azoles and allylamines, there are other relevant drug classes for the topical treatment of superficial mycoses. Butenafine, belonging to the benzylamines, is structurally closely related to allylamines (Figure 2; Table 2). Butenafine also inhibits squalene epoxidase, thereby interrupting ergosterol biosynthesis and causing toxic accumulation of squalene in the fungus, ultimately resulting in a fungicidal effect (Singal, 2008). Butenafine exhibits a broad antifungal spectrum, especially against dermatophytes, but also against *Aspergillus* species, dimorphic fungi, and dematiaceous fungi. Additionally, the drug has anti-inflammatory properties, which further enhance its clinical benefits (Singal, 2008). A mechanistically completely different drug class is the hydroxypyridones, with ciclopirox being the best-known representative. Ciclopirox exerts its effect through chelation of divalent metal ions (Figure 2; Table 2), thereby inactivating multiple metal-dependent fungal enzymes (Subissi et al., 2010; Niewerth et al., 2003). This enzyme inhibition disrupts essential cellular processes in fungi, resulting in a fungistatic effect. Besides its antifungal activity, ciclopirox also possesses antibacterial and anti-inflammatory properties. It is primarily used topically, especially for the treatment of onychomycosis in the form of medicated nail lacquer, but also for cutaneous mycoses in creams or ointments (Nenoff et al., 2015; Sahni et al., 2018). Another relevant drug class is the morpholines, represented by amorolfine. Amorolfine acts by inhibiting two enzymes involved in ergosterol biosynthesis, Δ14-reductase and Δ7–Δ8-isomerase (Figure 2; Table 2), leading both to depletion of ergosterol and accumulation of toxic intermediates as well as increased production of reactive oxygen and nitrogen species (Sahni et al., 2018; Carmo et al., 2022). This dual inhibition sustainably impairs fungal membrane integrity and results in fungicidal activity. Amorolfine shows high efficacy against dermatophytes, yeasts, and molds. It is used exclusively topically, primarily as a nail lacquer for the treatment of onychomycosis and in cream form for cutaneous mycoses (Nenoff et al., 2015; Sahni et al., 2018).

The increasing development of resistance among dermatophytes represents a growing global problem (Bristow and Joshi, 2023). Particularly concerning is the spread of the pathogen *T. indotineae*, which exhibits high resistance to terbinafine. The main causes are the uncontrolled distribution of antifungals, often combined with glucocorticoids, as well as inadequate long-term treatments lasting months to years (Bristow and Joshi, 2023). This improper use masks clinical symptoms, delays diagnosis, and promotes the selection of resistant strains. In Germany, resistant infections predominantly affect immunosuppressed patients and travelers returning with imported *T. indotineae* infections. Furthermore, there is a hitherto little-recognized risk of resistance development in zoophilic dermatophytes due to their introduction into agriculture and intensive livestock farming, with the possibility of retransmission to humans. Against this background, microbiological diagnostics prior to the start of therapy are essential to tailor drug selection to the individual resistance profile. In cases of suspected resistant pathogens, targeted antifungal therapy considering the resistance profile is imperative. If terbinafine resistance is present, alternative systemic agents such as itraconazole or fluconazole are indicated (Gupta et al., 2023a). Additionally, a combination of systemic and topical therapy, e.g., with clotrimazole or ciclopirox, should be employed to improve therapeutic outcomes.

A successful treatment should not only achieve a high cure, but also a low relapse rate. Ideally, it acts quickly, exhibits strong anti-inflammatory effects, has few side effects, minimal systemic absorption, and is cost-effective as well as safe during pregnancy, breastfeeding, and in patients with liver or kidney insufficiency (Jartarkar et al., 2021). In addition to pharmacological therapy, supportive measures play a crucial role. These include wearing loose-fitting cotton clothing, avoiding occlusive footwear, washing clothes more frequently, avoiding sharing bedding, towels, clothing, and shoes, and the regular application of medications (Jartarkar et al., 2021). Furthermore, discontinuation of corticosteroid-containing creams and screening of potential carriers, such as family members or pets, is recommended (Sahni et al., 2018).

## 5 Use of new antifungals, innovative formulations, and targeted combinations

Despite medical advances, therapeutic options for dermatophytoses remain limited. Currently, eight classes of antifungals are clinically available, of which only four are used in the treatment of dermatomycoses (Gnat et al., 2020). The eukaryotic cell structure of fungi complicates the development of selective agents, as substances must effectively inhibit fungi without harming the human host (Gnat et al., 2020). Additionally, topically applied antifungals must be able to penetrate keratinized surfaces such as skin and nails and remain bioavailable there (Monod et al., 2019; Gnat et al., 2020). The currently insufficient penetration capacity of many topical preparations often leads to sublethal drug concentrations at the site of action, which can promote the development of resistance (Gnat et al., 2020). Innovative drug formulations such as SUBA- or Meltrex-technologies improve oral bioavailability, for example, of itraconazole, while modern carrier systems like hydroxypropylchitosan (HPCH), transfersomes, or microsponge systems can enhance penetration through skin and nails (Poddar et al., 2023). These new formulations improve efficacy and patient adherence through shorter treatment durations and better penetration (Poddar et al., 2023; Sugiura et al., 2021). However, robust data correlating *in vitro* determined minimal inhibitory concentrations (MIC) with *in vivo* achievable drug levels are lacking (Sahni et al., 2018; Kaul et al., 2017). Due to rapidly progressing resistance development, the pace of which exceeds the development of new therapeutic approaches, there is an urgent need to integrate new or previously underutilized agents into clinical practice.

Newer triazoles such as posaconazole and voriconazole (Figure 2; Table 2), which exhibit strong *in vitro* activity against dermatophytes, are currently approved only for certain invasive systemic mycoses. Clinical data for dermatomycoses and onychomycoses are still lacking, thus these broad-spectrum triazoles have not yet received approval from the European Medicines Agency (EMA) or the FDA for the treatment of dermatomycoses or onychomycoses and can only be used off-label (Gupta et al., 2023a). Efinaconazole is a topical triazole (Figure 2; Table 2) specifically developed for the treatment of onychomycosis. Due to its low surface tension and lack of affinity for the nail plate, it penetrates the infected nail bed more effectively than other topical antifungals such as amorolfine or ciclopirox (Sugiura et al., 2021; Lipner and Scher, 2015). The imidazole luliconazole (Figure 2; Table 2), an R-enantiomer of lanoconazole, showed very strong *in vitro* activity against dermatophytes with the lowest minimal inhibitory concentrations (MIC), even surpassing terbinafine and ciclopirox (Wiederhold et al., 2014). Clinically, luliconazole is applied topically and is well tolerated (Watanabe et al., 2017; Jarratt et al., 2013). Tavaborole is not an azole but belongs to the benzoxaboroles, a new class of antifungal agents. It acts by inhibiting aminoacyl-tRNA synthetase (Figure 2; Table 2), thereby blocking fungal protein biosynthesis (Rich et al., 2019; Elewski et al., 2015). Tavaborole is also recommended topically for onychomycosis. Similar to efinaconazole, it exhibits very good penetration of the nail plate (Sugiura et al., 2021). Additionally, both efinaconazole and tavaborole have shown promising efficacy against otherwise resistant dermatophytes (Kruithoff et al., 2023). Further advances in functional genomics, proteomics, and genome mapping are crucial to identifying new cellular targets for innovative antifungals, such as the glyoxylate cycle, pyrimidine and heme biosynthesis, cytochrome P450 metabolism, iron metabolism, and signal transduction pathways like MAP and the calcineurin pathway (Gnat et al., 2020).

Combination therapies with topical and oral antifungals can be considered in cases of clinical treatment failures and represent a possible approach to prevent multidrug-resistant strains (Gupta et al., 2023a). Synergies have been demonstrated *in vitro* for terbinafine plus efinaconazole as well as for itraconazole plus efinaconazole, luliconazole, and tavaborole against various dermatophyte strains (Sugiura et al., 2021). Due to potential side effects, laboratory monitoring before and during therapy can be recommendable (Gupta et al., 2023a).

Another therapeutic approach involves combining classical antifungals with efflux pump inhibitors. Studies show that the efficacy of itraconazole and voriconazole against *T. rubrum* and *T. mentagrophytes* can be significantly enhanced *in vitro* by inhibiting MDR and MFS transporters (Günther et al., 2025; Gnat et al., 2021; Monod et al., 2019). The targeted use of such combinations could be a promising strategy to markedly improve effectiveness in resistant infections in the future.

## 6 Antifungal stewardship

Successful treatment of dermatophyte infections requires an individualized, resistance-aware therapeutic approach that considers local epidemiology, the infection pattern, and specific risk factors, especially in cases of onychomycosis, multidrug-resistant pathogens, or immunocompromised patients. Within the framework of antifungal stewardship (AFS), measures for the rational use of antifungals should be established. These include structured patient education, standardized laboratory diagnostic procedures, surveillance programs for detecting resistant pathogens, and evidence-based treatment recommendations based on current resistance data (Gupta et al., 2023a).

In principle, antifungal therapy for superficial fungal infections should be limited to cases in which fungal elements have been detected through appropriate diagnostics, thereby avoiding unnecessary drug exposure and potential treatment risks. Since the efficacy of antifungal agents can vary depending on the causative organism, identification of the pathogen at least to the genus level and ideally to the species level is recommended to select the optimal antifungal agent. If clinical treatment does not achieve sufficient success, repeat sampling and diagnostics must be considered.
